# ZO-2/Tjp2 suppresses Yap and Wwtr1/Taz-mediated hepatocyte to cholangiocyte transdifferentiation in the mouse liver

**DOI:** 10.1038/s41536-022-00251-6

**Published:** 2022-09-23

**Authors:** Jianliang Xu, P. Jaya Kausalya, Alicia Ghia Min Ong, Christine Meng Fan Goh, Safiah Mohamed Ali, Walter Hunziker

**Affiliations:** 1 Epithelial Polarity in Disease and Tissue Regeneration Laboratory, Institute of Molecular and Cell Biology, Agency for Science, Technology and Research (A*STAR), 61 Biopolis Drive Proteos, Singapore, 138673 Singapore; 2 Department of Physiology, Yong Loo Lin School of Medicine, National University of Singapore, 2 Medical Drive MD9, Singapore, 117593 Singapore; 3 Present Address: M Diagnostics Pte. Ltd. (MiRXES), 30 Biopolis Road, #09-05/06 Matrix, Singapore, 138671 Singapore

**Keywords:** Transdifferentiation, Cell signalling

## Abstract

*TJP2/ZO-2*-inactivating mutations in humans cause progressive cholestatic liver disease. Liver-specific deletion of *Tjp2* in the mouse (*Tjp2* cKO mice) leads to mild progressive cholestasis without an overt degradation of the bile-blood barrier (BBB). These mice are more susceptible to cholic acid (CA) induced liver injury. Interestingly, while initially also more susceptible, *Tjp2* cKO mice develop tolerance to a DDC-supplemented diet. The DDC diet induces an exacerbated ductular reaction in *Tjp2* cKO mice, which arises from the transdifferentiation of hepatocytes to cholangiocytes. Consequently, this transdifferentiation is only observed if *Tjp2* is inactivated in hepatocytes, but not if deleted in cholangiocytes. The DDC-diet-induced hepatocyte transdifferentiation in *Tjp2* cKO mice requires *Yap* and *Wwtr1/Taz*, whose protein expression is upregulated in hepatocytes lacking *Tjp2*, but is independent of Notch2. Although inactivating *Tjp2* is sufficient for the upregulation of Yap and Wwtr1/Taz protein, efficient transdifferentiation requires the DDC-diet insult. Thus, Tjp2 negatively regulates Yap/Taz-mediated transdifferentiation of hepatocytes to cholangiocytes in response to DDC-diet-induced liver injury. Furthermore, transdifferentiation is regulated at multiple levels and the type of injury inflicted on the *Tjp2* deficient liver plays an important role in the resulting pathophysiology.

## Introduction

The liver plays key roles in the metabolism of amino acids, lipids, and carbohydrates, as well as in the production and excretion of bile. Hepatocytes carry out metabolic and detoxification reactions and produce bile. Bile acids are excreted into the canaliculi, from where the bile drains into intrahepatic bile ducts lined by bile duct epithelial cells or cholangiocytes, from where it is delivered to the gall bladder via the extrahepatic bile duct. Tight junctions (TJs) between adjacent hepatocytes seal the canaliculus to form the bile-blood barrier, which restricts the diffusion of bile from the canaliculus to the serosal tissue compartment.

As a major metabolic detoxification hub, the liver is continuously exposed to bile acids and other cytotoxic chemicals. These insults commonly result in increased plasma bile acid levels, reduced liver function, and expansion of bile duct epithelial cells (e.g. a ductular reaction) and, if chronic, ultimately lead to liver fibrosis and cirrhosis. The organ has developed a remarkable array of regenerative strategies to cope with injury. Hepatocytes can proliferate and reconstitute a significant mass of the damaged tissue. If hepatocyte proliferation is insufficient, still poorly characterized liver progenitor cells (known as oval cells in the mouse) are activated to generate cholangiocytes and hepatocytes. DDC-diet-induced liver injury activates the transdifferentiation of hepatocytes into cholangiocytes, a process that is regulated by the Notch^1^ and Hippo^2^ signaling pathways.

TJs of the BBB are composed of different integral transmembrane proteins, including members of the claudin (for example, Cldn1) and MARVEL (for example, Ocln) protein families. Cytosolic adaptor proteins such as ZO-1/Tjp1 and ZO-2/Tjp2 tether these transmembrane proteins to the underlying actomyosin cytoskeleton. In addition to their structural roles, Tjp1and Tjp2, act as hubs to regulate major signaling pathways. Through their multiple protein-protein interaction domains they associate with and modulate, often in response to cell–cell contact cues, different signaling factors, and transcriptional regulators. Tjp1 or Tjp2, for example, have been implicated in regulating the cytosolic/nuclear localization of the Hippo pathway transcriptional effectors Yap and Wwtr1/Taz^3–8^.

*TJP2*-inactivating familial mutations in humans are associated with progressive cholestasis^9–12^. The role of Tjp2 in liver biology has been explored using mouse models^13,14^ as well as liver organoids derived from *Tjp2* knock-out or patient-specific induced pluripotent stem cells^15^. While mice lacking *Tjp2* are embryonic lethal^16^, the liver-specific inactivation results in mild progressive cholestasis^14^ without an overt defect in the BBB^13,14^. *Tjp2* deficiency presents with a redistribution of radixin and less canalicular microvilli^14,15^, previously linked to cholestasis^17^, as well as changes in bile acid transporter and detoxification enzyme expression and distribution^13–15^.

Here, we characterized the effect of a DDC diet on *Tjp2* cKO mice. Interestingly, *Tjp2* cKO mice develop tolerance to prolonged DDC-diet feeding, likely due to the increased formation of duct-like structures that facilitate the clearance of bile. This DDC-diet-induced ductular reaction arises from an enhanced transdifferentiation of hepatocytes to cholangiocytes, which requires Yap/Wwtr1 and is negatively modulated by Tjp2.

## Results

### Liver-specific inactivation of *Tjp2* leads to an exacerbated ductular reaction in response to DDC-diet

**Fig. 1:**
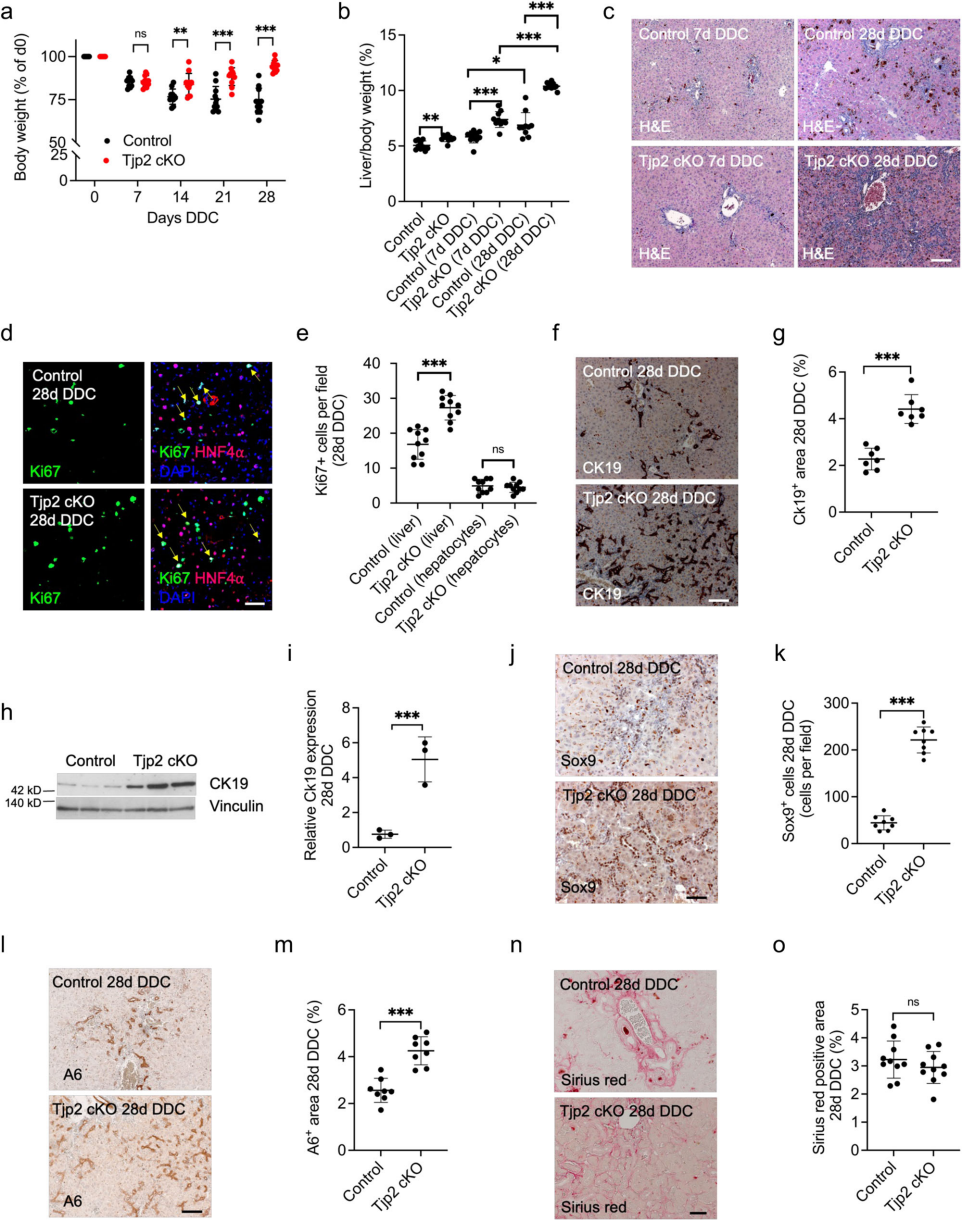
DDC diet induces a strong ductular reaction in the *Tjp2* cKO mouse liver. **a** Change of relative body weight for control and *Tjp2* cKO mice fed DDC diet for 28 days. **b** Liver to body weight ratio of indicated mouse lines after 7- and 28-day DDC-diet. **c** H&E staining for control and *Tjp2* cKO mouse liver after 7- and 28-day DDC-diet. Scale bar: 100 μm. **d**, **e** Immunofluorescence microscopy and quantification of Ki67-positive cells and Ki67-positive HNF4α-positive hepatocytes in the liver of mice fed DDC diet for 28 days. Scale bar: 50 μm. **f**, **g** Immunohistochemistry staining for Ck19 showing the periportal area and quantification of the Ck19-positive liver area from random liver sections after a 28-day DDC-diet. Scale bar: 100 μm. **h**, **i** Western blot analysis and quantification of Ck19 expression in control and *Tjp2* cKO mouse liver samples after a 28-day DDC diet. **j**, **k** Immunohistochemistry staining and quantification of Sox9-positive cells after a 28-day DDC-diet. Scale bar: 100 μm. **l**, **m** Immunohistochemistry staining and quantification of A6-positive liver area after a 28-day DDC diet. Scale bar: 100 μm. **n**, **o** Sirius red staining and quantification of Sirus red-positive liver area after a 28-day DDC diet. Scale bar: 50 μm. Data in (**a**, **b**, **e**, **g**, **i**, **k**, **m**, **o**) shown as mean ± SD, unpaired Student’s *t* test. *p < 0.05; **p < 0.005, ***p < 0.0005, ns—not significant (*p* > 0.05), with *p* < 0.05 considered a significant difference. (**a**, b, **e**, **o**): *n* = 10, (**g**): *n* = 7, (**k**, **m**): *n* = 8, (**i**) *n* = 3 mice per cohort, data from blot (**h**).

The body weight of control or *Tjp2* cKO mice fed normal chow supplemented with 0.1% DDC (DDC-diet) gradually declined (Fig. 1a). In contrast to controls, however, the body weight of *Tjp2* cKO animals fed DDC diet stabilized after week 2 and then partially recovered, suggesting that these mice had adapted to cope with the effect of the DDC-diet. Liver-to-body weight ratios were not only significantly higher in *Tjp2* cKO mice compared to controls, but the increase between 7 and 28 days of DDC diet was also more pronounced in the absence of hepatic *Tjp2* (Fig. 1b). While the histology of control and *Tjp2* cKO livers were comparable after 7 days of DDC diet (Fig. 1c), cell proliferation, as shown by more Ki67-positive cells detected by immunofluorescence microscopy, was enhanced in livers of *Tjp2* cKO mice fed DDC diet for 28 days (Fig. 1d, e). Interestingly, however, the number of Ki67-positive hepatocytes, identified by Hnf4α staining, was indistinguishable between controls and *Tjp2* cKO livers. Similar results were obtained by using Edu incorporation to monitor cell proliferation (Supplementary Fig. 1a, b), pointing to the expansion of a non-hepatocyte cell pool in the liver of *Tjp2* cKO mice fed DDC-diet. The number of Hnf4α-positive hepatocytes per liver area remained similar (Supplementary Fig. 1c, d) and no difference in cell or hepatocyte proliferation was observed between livers of control and Tjp2 cKO mice fed a regular chow (Supplementary Fig. 1e, f).

As expected, after 28 days of DDC-diet, the control livers presented with a ductular reaction, characterized by tubular structures in the portal region (Fig. 1f). In the *Tjp2* cKO liver, these Ck19 ductular structures did not remain localized to the portal area but were found further in the liver parenchyma, as shown by a larger Ck19-positive periportal area (Fig. 1g). Immunohistochemistry staining and quantification for Sox9 (Fig. 1j, k) and the oval cell marker A6 (Fig. 1l, m) confirmed the ductular nature of these cells and their higher abundance and broader distribution in the liver of DDC-diet-fed *Tjp2* cKO mice. Consistent with the ductular reaction and the larger Ck19-positive area, Ck19 expression, assessed by Western blot (Fig. 1h, i) or qRT-PCR (Supplementary Fig. 1g), was highly increased in livers of mice fed DDC-diet for 28 days. mRNA expression of Epcam and Osteopontin, two genes involved growth of cholangiocytes, was strongly increased in DDC-diet-fed mice, and more so in Tjp2 cKO animals as compared to controls. While the fraction of fibrotic tissue in control and *Tjp2* deficient livers was comparable (Fig. 1n, o), Sirius red staining intensity, restricted to the portal area in controls, was reduced in *Tjp2* cKO livers but distributed throughout the whole liver tissue (Fig. 1n). Despite the different pattern of Sirius red staining, qRT-PCR and Western blot analysis and quantification of different fibrosis markers confirmed the comparable fibrosis in control and *Tjp2* cKO mouse livers (Supplementary Fig. 1h–k). Abundant DDC-mediated porphyrin deposition was seen in control liver sections, but significantly less so in livers lacking *Tjp2* (Fig. 1c), likely due to reduced protein levels of Alas-1, the rate limiting enzyme in porphyrin synthesis (Supplementary Fig. 1l–o).

### Mice with hepatic deletion of *Tjp2* develop tolerance to the DDC-diet

**Fig. 2:**
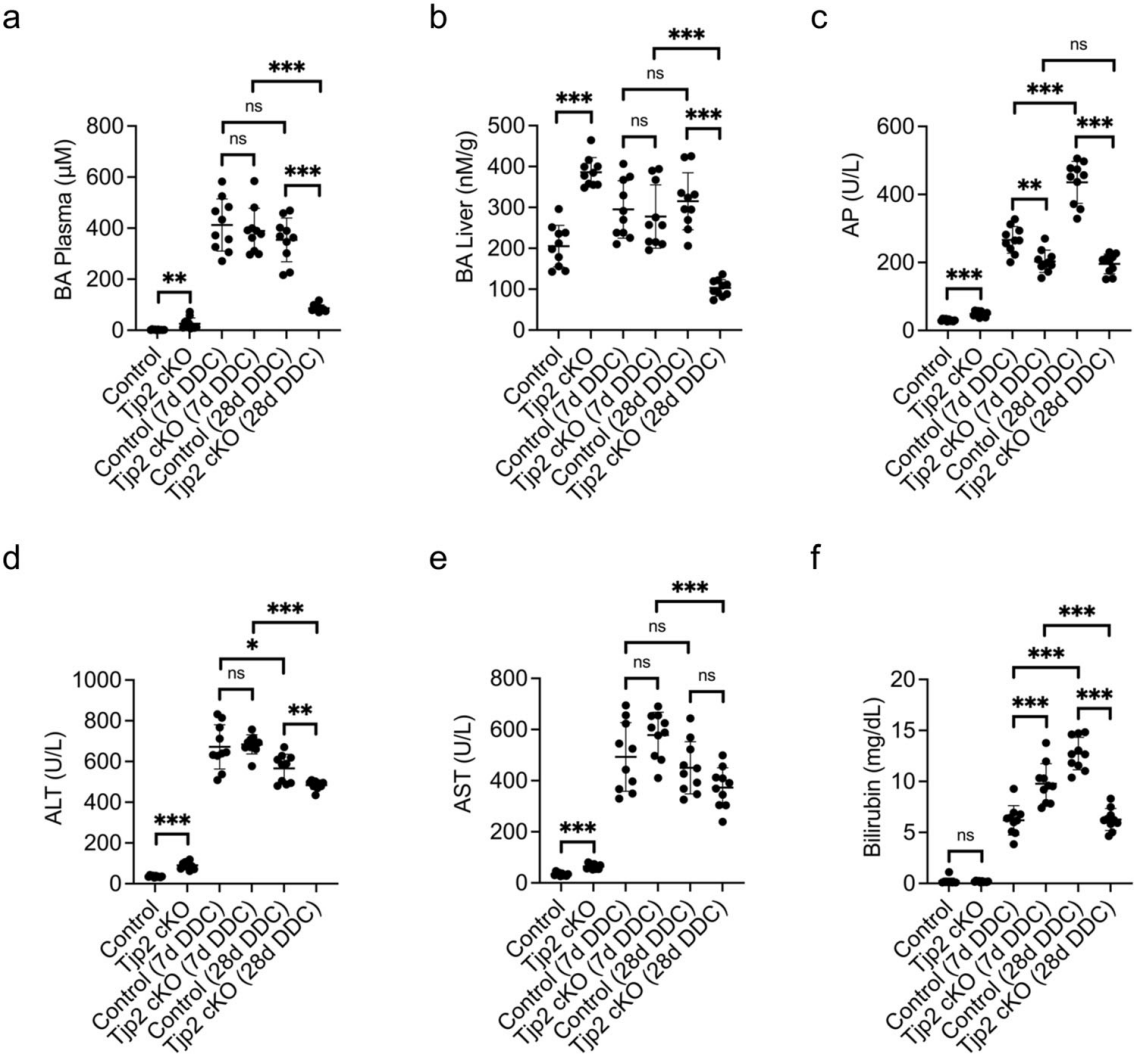
Hepatic deletion of *Tjp2* suppresses DDC-diet-induced liver injury. Biochemical analysis of liver and plasma markers for control and *Tjp2* cKO mice fed DDC diet for 7 and 28 days. **a** Plasma BA, (**b**) liver BA, (**c**) AP, (**d**) ALT, (**e**) AST, (**f**) Bilirubin. Data shown as mean ± SD, unpaired Student’s *t* test. *p < 0.05; **p < 0.005, ***p < 0.0005, ns—not significant (*p* > 0.05), with *p* < 0.05 considered a significant difference. *n* = 10 mice per cohort.

Tjp2 cKO mice show progressive cholestasis (Fig. 2a–e and^14^) and are more susceptible to a CA-diet^14^. In animals fed the DDC diet for 7 days, blood and liver biochemistry parameters (e.g., BA, AP, ALT, AST, and bilirubin levels) were elevated as compared to control chow-fed cohorts, but, except for AP and bilirubin, comparable for DDC-diet fed *Tjp2* cKO and control mice (Fig. 2). However, by 28 days, while BA levels remained elevated in controls, they were significantly lower in mice lacking hepatic *Tjp2* (Fig. 2a, b). Cholestasis severity, assessed by AP levels, was lower in DDC-diet-fed *Tjp2* cKO mice and did not worsen with time as compared to corresponding control animals (Fig. 2c). Compared to corresponding controls, *Tjp2* cKO mice also showed milder DDC-diet-induced liver injury, with ALT levels significantly lower (Fig. 2d) and AST (Fig. 2e) levels trending lower at 28 days of DDC-diet feeding. Liver function, monitored by bilirubin levels, was initially more severely affected by the DDC diet in *Tjp2* cKO mice (Fig. 2f). However, while liver function continued to deteriorate in controls, it improved in *Tjp2* cKO animals by 28 days of DDC-diet feeding, consistent with the lower BA levels and liver injury. Thus, while more susceptible to a CA-diet^14^, *Tjp2* cKO mice develop tolerance to chronic DDC-diet feeding, suggesting that the type of liver injury influences the pathophysiology in these mice.

### Deletion of *Tjp2* in hepatocytes, not cholangiocytes, mediates the enhanced DDC-diet-induced ductular reaction in the *Tjp2* cKO liver

*Tjp2* is inactivated in both hepatocytes and cholangiocytes of *Tjp2* cKO mice^14^. To determine whether the enhanced DDC-induced ductular reaction in these animals was due to the absence of *Tjp2* from a particular cell type, we took advantage of mice where *Tjp2* can be inducibly-inactivated in cholangiocytes (*Tjp2* icKO^CC^) or hepatocytes (*Tjp2* icKO^HC^)^14^.

**Fig. 3:**
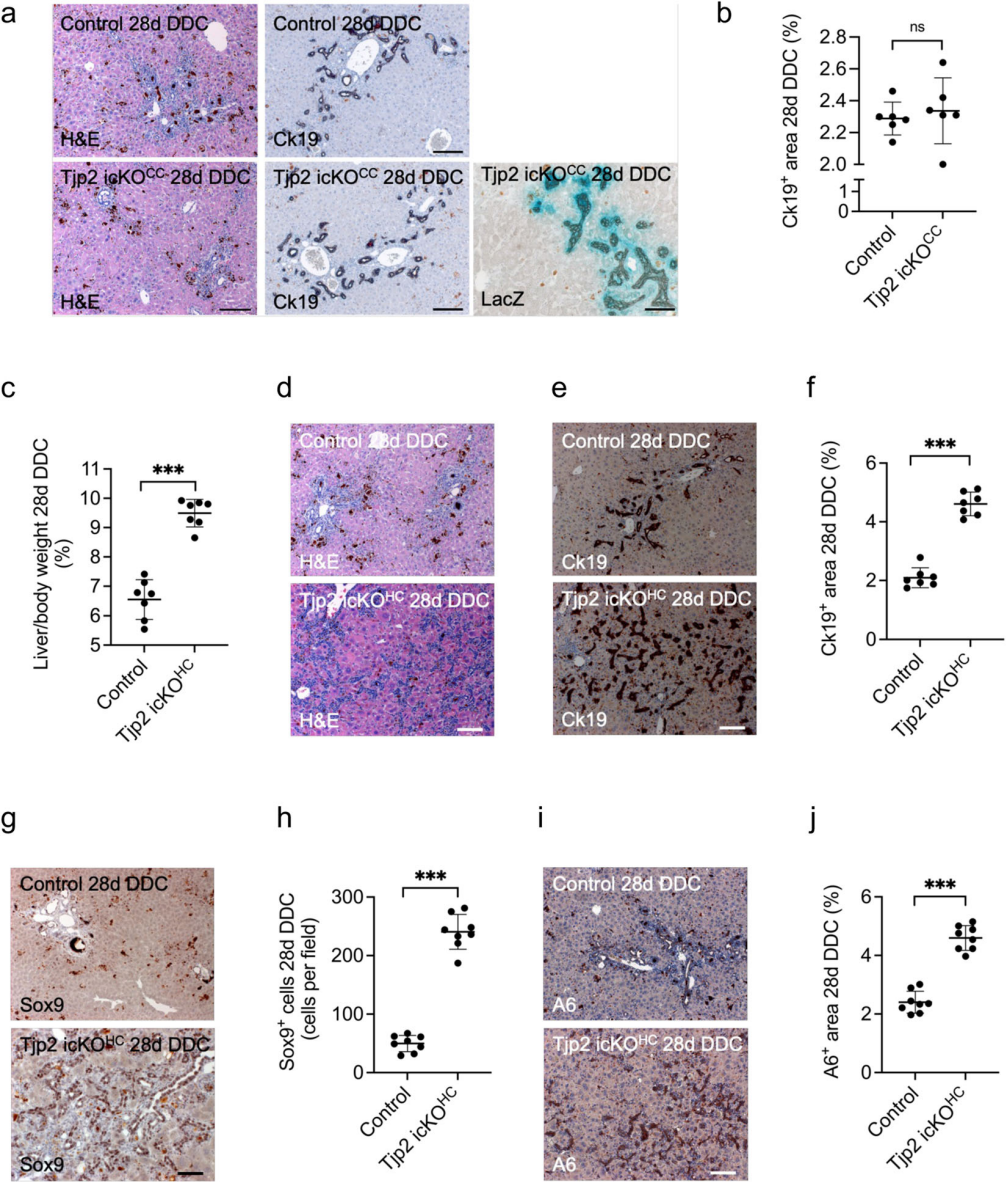
Deletion of *Tjp2* in hepatocytes, but not in cholangiocytes, leads to a strong ductular reaction after DDC-diet. **a** H&E, Ck19, and LacZ staining of liver sections from control and *Tjp2* icKO^CC^ mice after a 28-day DDC-diet. Scale bar: 100 μm. **b** Quantification of the Ck19-positive liver area. **c** Liver to body weight ratio for control and *Tjp2* icKO^HC^ mice after a 28-day DDC-diet. **d** H&E staining for control and *Tjp2* icKO^HC^ mice after a 28-day DDC-diet. Scale bar: 100 μm. **e**, **f** Ck19 staining showing periportal areas from control and *Tjp2* icKO^HC^ mice after a 28-day DDC diet and quantification from random liver sections. Scale bar: 100 μm. **g**, **h** Sox9 staining of liver sections from control and *Tjp2* icKO^HC^ mice after a 28-day DDC diet and quantification. Scale bar: 100 μm. **i**, **j** A6 staining of liver sections from control and *Tjp2* icKO^HC^ mice after a 28-day DDC diet and quantification. Scale bar: 100 μm. Data in (**b**, **c**, **f**, **h**, **j**) shown as mean ± SD, unpaired Student’s *t* test. *p < 0.05; **p < 0.005, ***p < 0.0005, ns = not significant (*p* > 0.05), with *p* < 0.05 considered a significant difference. (**b**): *n* = 6, (**c**, **f**): *n* = 7, (**h**, **j**): n-8 mice per cohort. icKO^HH^ or icKO^CC^, tamoxifen-induced hepatocyte or cholangiocyte deletion, respectively.

As assessed by H&E staining, *Tjp2* icKO^CC^ mice fed the DDC diet for 28 weeks showed similar liver histology as controls (Fig. 3a). Immunohistochemical staining for Ck19 and quantification (Fig. 3a, b) showed no difference in the extent of ductular reaction in *Tjp2* icKO^CC^ livers compared to controls. Staining for the LacZ reporter confirmed efficient activation of Cre recombinase in cholangiocytes, with most of the ductular reaction originating from these LacZ-positive cholangiocytes.

In contrast, *Tjp2* icKO^HC^ mice fed the DDC diet presented with a higher liver-to-body weight ratio (Fig. 3c). H&E staining revealed duct-like structures further into the liver parenchyma (Fig. 3d), like what was observed in *Tjp2* cKO livers. Immunohistochemistry staining for Ck19 (Fig. 3e, f), Sox9 (Fig. 3g, h), and A6 (Fig. 3i, j) confirmed that the cells of these duct-like structures expressed cholangiocyte markers.

### Conditional inactivation of *Tjp2* facilitates DDC-diet-induced transdifferentiation of hepatocytes to cholangiocytes

**Fig. 4:**
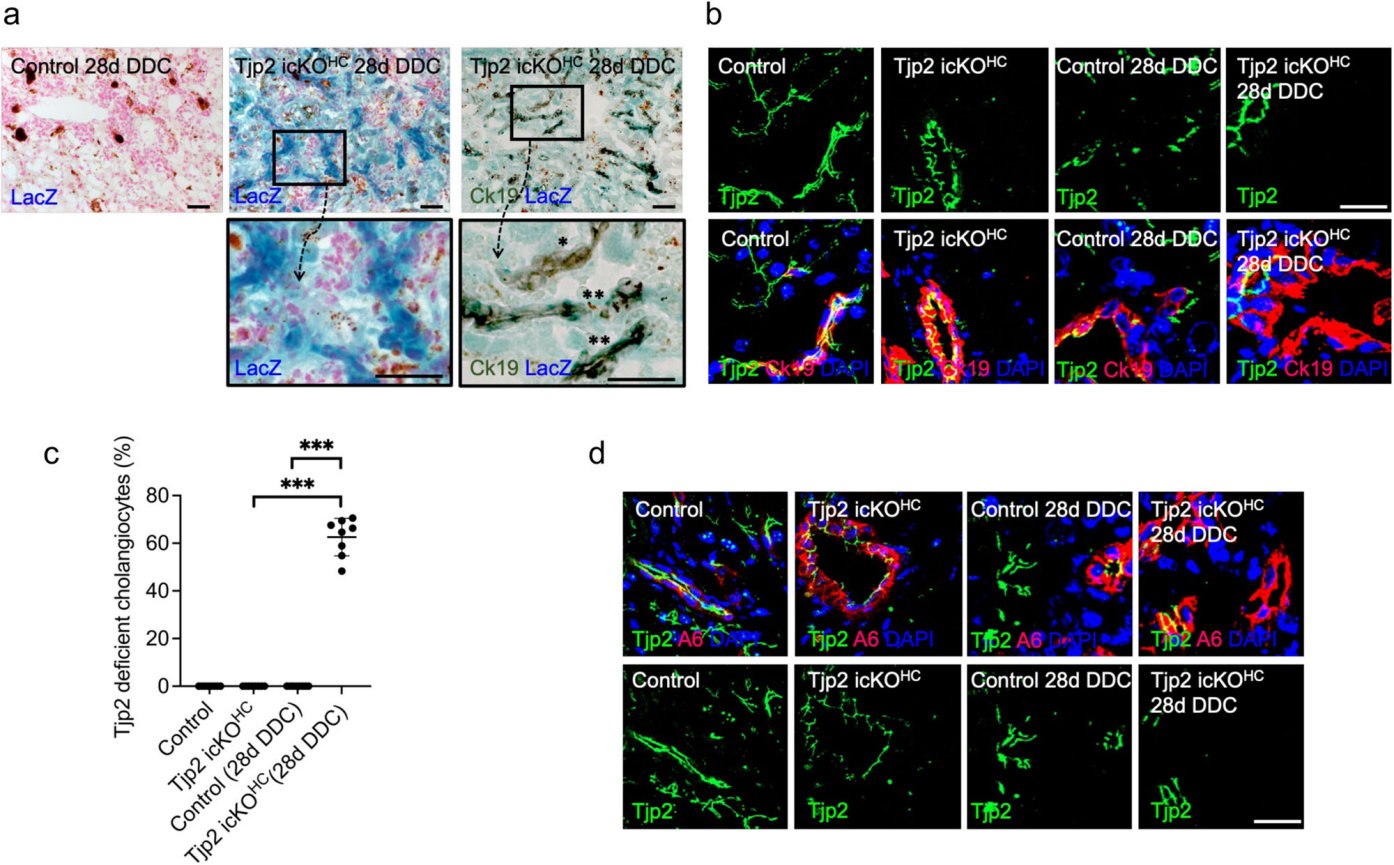
*Tjp2* deficient hepatocytes give rise to the DDC-diet-induced ductular cells. **a** Lineage tracing. One week after the last tamoxifen dose to induce deletion of the indicated genes, mice were fed a DDC diet for 28 days and sacrificed. LacZ and Ck19 staining of control and *Tjp2* icKO^HC^ Rosa26:LacZ mouse liver samples, showing Ck19-positive ductular structures derived from LacZ-positive hepatocytes (✽✽) and resident LacZ-negative Ck19-positive bile ducts (✽). Scale bar: 50 μm. **b**, **c** Tjp2 and Ck19 staining of control and *Tjp2* icKO^HC^ liver sections from mice fed standard or DDC diet and quantification. Scale bar: 20 μm. **d** Tjp2 and A6 staining of control and *Tjp2* icKO^HC^ liver sections from mice fed standard or DDC-diet. Note that in *Tjp2* icKO^HC^ animals, *Tjp2* is deleted in hepatocytes, thus showing that Tjp2-negative and Ck19- or A6-positive ductular cells arise from *Tjp2*-deficient hepatocytes. icKO^HH^, tamoxifen-induced hepatocyte deletion. Scale bar: 20 μm.

Several recent studies have established that hepatocytes can transdifferentiate into cholangiocytes^18,19^. Since hepatocyte deletion of *Tjp2* led to the DDC-induced expansion of cholangiocyte-like cells throughout the liver tissue, we tested if these cholangiocytes were derived from transdifferentiated hepatocytes, using tamoxifen-treated Tjp2^F/F^ AlbCre^ERT2^ Rosa26:Lox-STOP-Lox-LacZ mice^14^ for lineage tracing. One week after the tamoxifen regime, the resulting control Rosa26:LacZ or *Tjp2* icKO^HC^ Rosa26:LacZ mice were fed the DDC diet for 28 days before the collection and analysis of livers. Cre was selectively expressed in hepatocytes as shown by LacZ expression in Ck19-negative cells. In livers of *Tjp2* icKO^HC^ Rosa26:LacZ mice, the duct-like structures showed intense blue LacZ staining, with many of the strong LacZ-positive cells also labeling for Ck19 (Fig. 4a). Hepatocytes with a fainter LacZ staining surrounded these intensely labeled cells.

In the *Tjp2* icKO^HC^ liver, Tjp2 expression is inactivated in hepatocytes but not in cholangiocytes^14^. Thus, if the expanded bile duct-like structures induced by DDC-diet feeding arise from *Tjp2* deficient hepatocytes, they should also carry an inactivated *Tjp2* gene. Bile ducts in livers of control or *Tjp2* icKO^HC^ mice fed standard chow showed, as expected, Tjp2-positive bile ducts (Fig. 4b). While bile ducts were also Tjp2-positive in control animals fed the DDC diet for 28 days, Tjp2 was absent from most of the Ck19- or A6-positive duct-like structures in *Tjp2* icKO^HC^ livers (Fig. 4b–d), corroborating the LacZ lineage tracing experiment. The data thus indicates that following inactivation of *Tjp2* in hepatocytes, the DDC diet induces a strong ductular reaction, whereby new cholangiocytes arise by transdifferentiation of hepatocytes.

### Increased protein levels and nuclear localization of Yap/Taz in hepatocytes lacking *Tjp2*

**Fig. 5:**
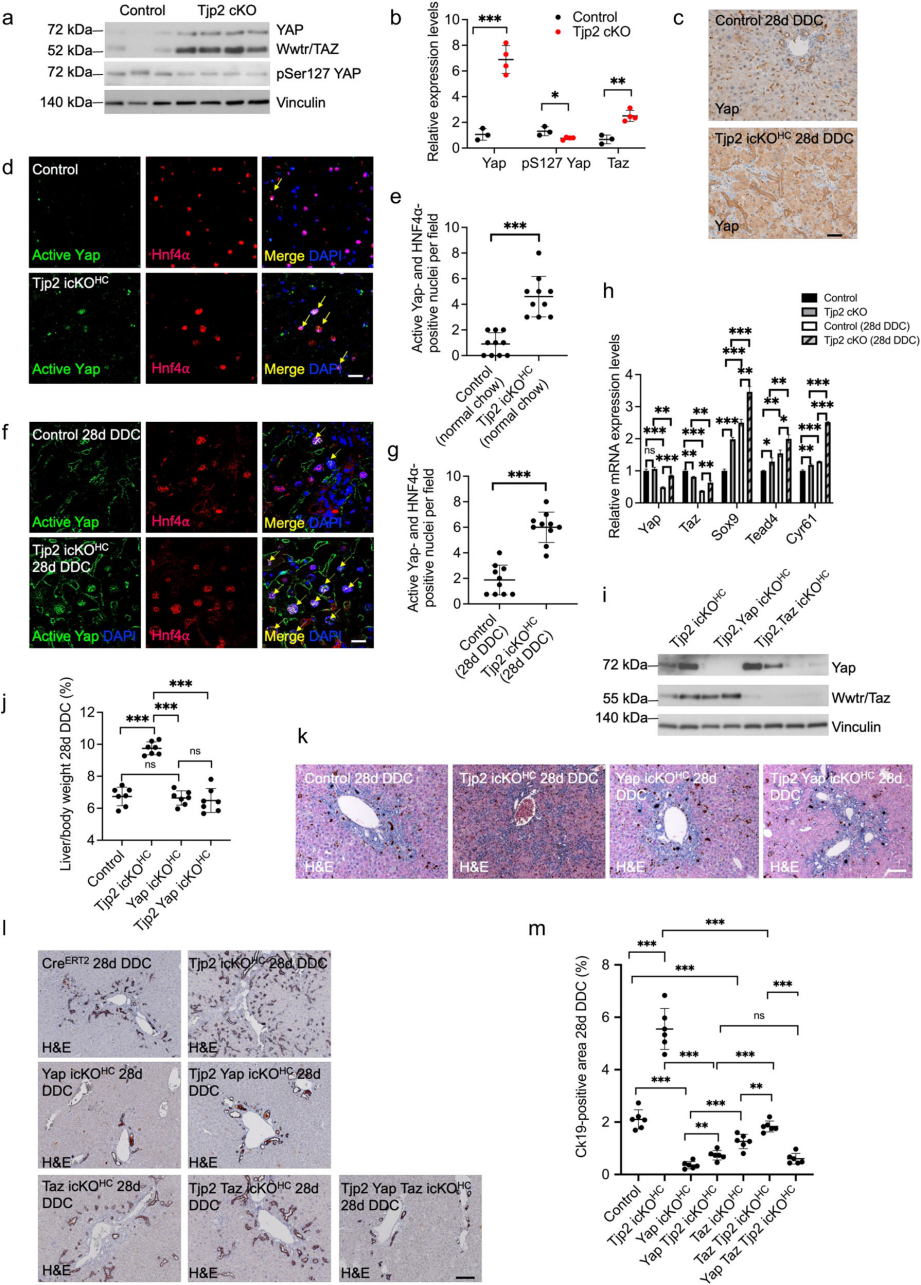
DDC-diet-induced ductular reaction in *Tjp2* cKO mice requires *Yap*/*Taz*. **a**, **b** Western blot analysis and quantification of Yap, Taz and pSer127 Yap expression in isolated hepatocytes from control and *Tjp2* cKO mice fed normal chow. **c** Immunohistochemistry staining for Yap in control and *Tjp2* icKO^HC^ liver sections after a 28-day DDC-diet. Scale bar: 50 μm. **d**, **f** Immunofluorescence labeling of active (e.g. not S127 phosphorylated) Yap and HNF4α, a nuclear hepatocyte marker in liver sections of mice fed a standard (**d**) or a DDC- supplemented (**f**) diet. Scale bar: 20 μm. **e**, **g** Quantification of Yap and HNF4α-positive nuclei. **h** Relative mRNA expression levels of key Hippo effectors and target genes in liver of control or *Tjp2* cKO mice fed normal or DDC-supplemented chow. **i** Western blot analysis of Yap and Wwtr1/Taz to verify their deletion in the liver of the corresponding mouse lines**. j** Liver to body weight ratios for the indicated mouse lines fed DDC diet for a 28-day period**. k** H&E staining of liver sections for the indicated mouse lines after a 28-day DDC-diet. Scale bar: 100 μm. **l**, **m** Immunohistochemistry staining for Ck19 in liver sections for the indicated mouse lines after a 28-day DDC diet and quantification of the Ck19-positive area. Scale bar: 100 μm. Data in (**b**, **e**, **g**, **h**, **j**, **m**) shown as mean ± SD, unpaired Student’s *t* test. *p < 0.05; **p < 0.005, ***p < 0.0005, ns=not significant (*p* > 0.05), with *p* < 0.05 considered a significant difference. (**b**): *n* = 3 or 4, (**e**) and (**g**): *n* = 10, (**h**): *n* = 3, (**j**): *n* = 7, (**m**): *n* = 6 mice per cohort. icKO^HH^, tamoxifen-induced hepatocyte deletion.

Overexpression of Yap in the liver induces transdifferentiation of hepatocytes to cholangiocytes^2^. Furthermore, Tjp2 associates with Yap and Taz and retains them in the cytosol, where they undergo degradation^4–7^. We therefore hypothesized that Tjp2 may suppress hepatocyte transdifferentiation via Yap and/or Taz. In hepatocytes isolated from control or *Tjp2* cKO mice fed a standard chow, total Yap and Taz protein expression levels were strongly increased in the latter as assessed by Western blot analysis (Fig. 5a, b). Interestingly, despite the increased expression level of Yap, pS127 Yap levels were reduced in the absence of *Tjp2*. Immunohistochemistry and immunofluorescence microscopy confirmed stronger Yap staining in liver sections of *Tjp2* icKO^HC^ mice fed standard chow or a DDC diet for 28 days as compared to corresponding controls (Fig. 5c, d, f). Since pS127 Yap is retained in the cytosol and subject to degradation by the proteasome pathway^20^, the reduced pS127 Yap level in *Tjp2* cKO hepatocytes is consistent with the observed higher total Yap protein levels and suggests enhanced Yap activity as compared to control hepatocytes. Indeed, while only a few cells showed nuclear colocalization of Yap and the hepatocyte marker Hnf4α^21^ in control mice, 3–5 times as many Hnf4α-positive hepatocytes showed nuclear localization of active (e.g. not phosphorylated on S127) Yap in *Tjp2* icKO^HC^ mice fed either the standard (Fig. 5d, e) or the DDC-supplemented (Fig. 5f, g) diet. Hepatocellular expression levels and nuclear staining intensity for active YAP were not only increased after deletion of *Tjp2* (Fig. 5a–e), but they were further increased by the DDC diet, both in controls and *Tjp2* icKO^HC^ mice (Fig. 5f, g). As expected, the expression of known Yap/Taz target genes, including Sox9 which promotes cholangiocyte fate, were upregulated after *Tjp2* deletion and/or DDC-diet feeding (Fig. 5h), confirming higher Yap activity. These data are consistent with the DDC diet and Tjp2 deletion influencing cellular YAP levels, with Tjp2 also playing a key role in negatively regulating Yap/Taz by retaining these Hippo pathway effectors in the cytosol of hepatocytes.

### *Yap* and *Taz* are required for DDC-diet-induced ductular reaction and suppression of liver injury in hepatic *Tjp2* deficient mice

To determine whether Yap/Taz are required for the DDC-diet-induced phenotype in *Tjp2* icKO^HC^ mice, we crossed Alb-Cre^ERT2^ Tjp2^F/F^ mice with *Yap*^F/F^, *Taz*^F/F^ or *Yap*^F/F^ *Taz*^F/F^ mice^22,23^, which after tamoxifen induction result in mice with an hepatocyte-inactivation of *Tjp2* and *Yap* (*Tjp2 Yap* icKO^HC^), *Tjp2* and *Taz* (*Tjp2 Taz* icKO^HC^) or *Tjp2*, *Yap* and *Taz* (*Tjp2 Yap Taz* icKO^HC^). Western blot analysis for the Hippo effectors confirmed their efficient inactivation in hepatocytes of the respective mouse strains (Fig. 5i).

While the initial decline in body weight of DDC-diet-fed *Tjp2* icKO^HC^ mice recovered from 7 days onward (Fig. 1a), this recovery was abrogated in *Tjp2 Yap* icKO^HC^ mice (Supplementary Fig. 2). Likewise, the liver-to-body weight ratio increase observed in *Tjp2* icKO^HC^ animals fed DDC diet for 28 days (Fig. 1b) was stunted in the absence of *Yap* (Fig. 5j).

H&E staining did not reveal duct-like structures in the livers of *Yap* icKO^HC^ or *Tjp2 Yap* icKO^HC^ mice fed DDC diet for 28 days, indicating that the ductular reaction observed in the *Tjp2* icKO^HC^ liver requires *Yap* (Fig. 5k). By immunohistochemistry, Ck19-positive bile duct-like structures, observed in livers from *Tjp2* icKO mice fed DDC diet for 28 days, were strongly reduced in *Tjp2 Yap* icKO^HC^ and, to a lesser extent, *Tjp2 Taz* icKO^HC^ tissues (Fig. 5k, l). Livers where either *Yap* or *Taz* was inactivated showed less Ck19-positive staining compared to controls and deletion of both *Yap* and *Taz* in the *Tjp2* deficient background further suppressed the ductular reaction (Fig. 5m). These data indicate that both Yap and Taz contribute to the DDC-diet-induced ductular reaction observed in *Tjp2* icKO mice. Given the more pronounced effect of inactivating *Yap*, we focused on Yap in subsequent experiments.

**Fig. 6:**
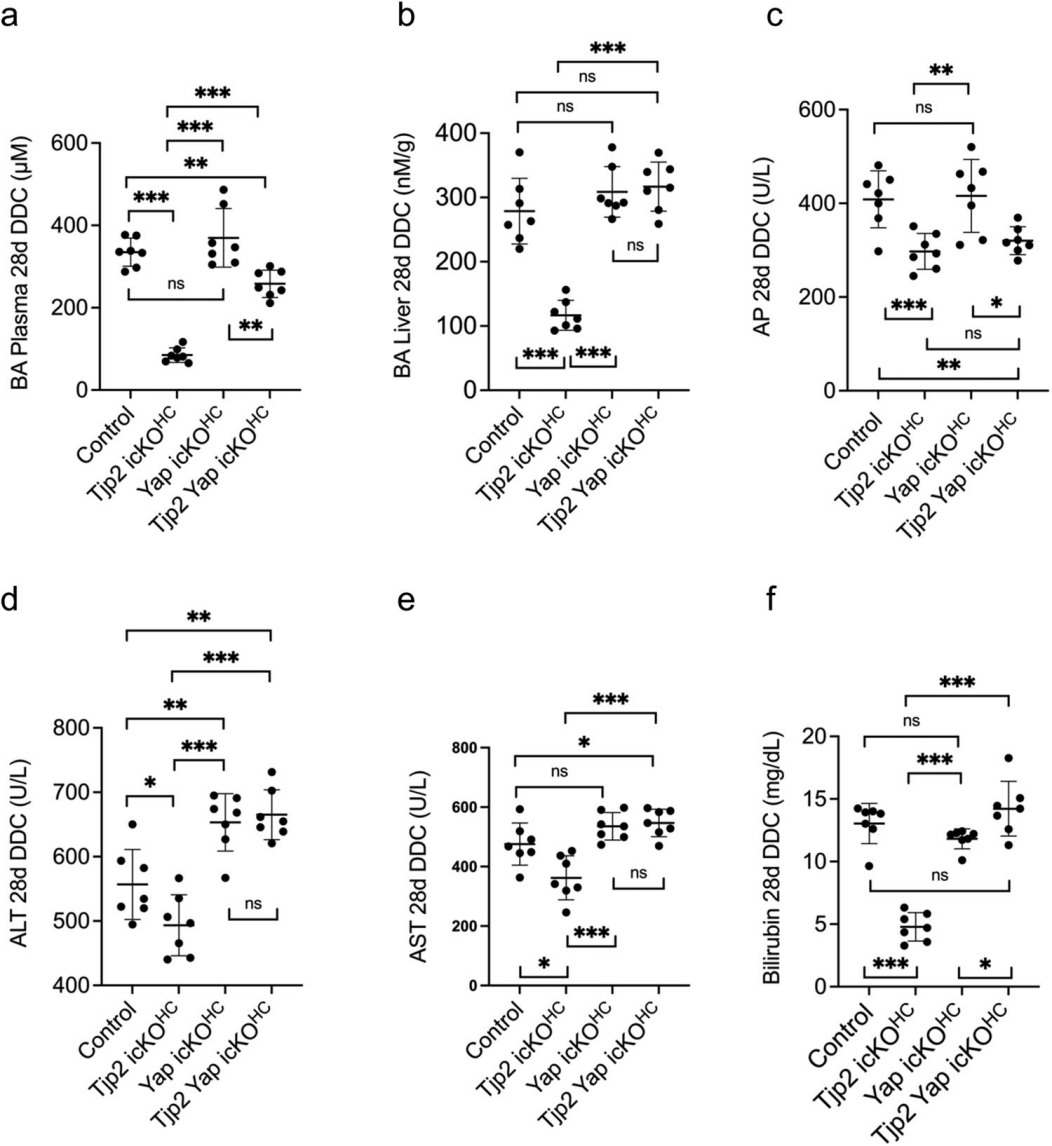
Biochemical analysis of liver and plasma marker levels for control, *Tjp2* icKO^HC^, *Yap* icKO^HC^, and *Tjp2 Yap* icKO^HC^ mice fed a 28-day DDC diet. **a** Plasma BA, (**b**) liver BA, (**c**) AP, (**d**) ALT, (**e**) AST, (**f**) Bilirubin. Data are shown as mean ± SD, unpaired Student’s *t* test. *p < 0.05; **p < 0.005, ***p < 0.0005, ns—not significant (*p* > 0.05), with *p* < 0.05 considered a significant difference. *n* = 7 mice per cohort. icKO^HH^, tamoxifen-induced hepatocyte deletion.

Overall, the improved DDC-diet-induced blood and liver biochemistry values observed in *Tjp2* icKO^HC^ mice compared to controls were abrogated if *Yap* was inactivated in *Tjp2* icKO^HC^ animals. Plasma (Fig. 6a) and liver (Fig. 6b) BA levels in *Tjp2 Yap* icKO^HC^ mice were increased compared to control mice. While AP levels were not significantly different between *Tjp2* icKO^HC^ and *Tjp2 Yap* icKO^HC^ mice (Fig. 6c), plasma ALT (Fig. 6d), AST (Fig. 6e), and bilirubin (Fig. 6f) were all elevated in the latter to levels comparable to controls. Thus, Yap is required for the development of tolerance to DDC-diet-induced liver injury observed in mice where *Tjp2* has been ablated in hepatocytes.

### DDC-diet-induced transdifferentiation of *Tjp2*-deficient hepatocytes into cholangiocytes requires Yap and is independent of Notch2

**Fig. 7:**
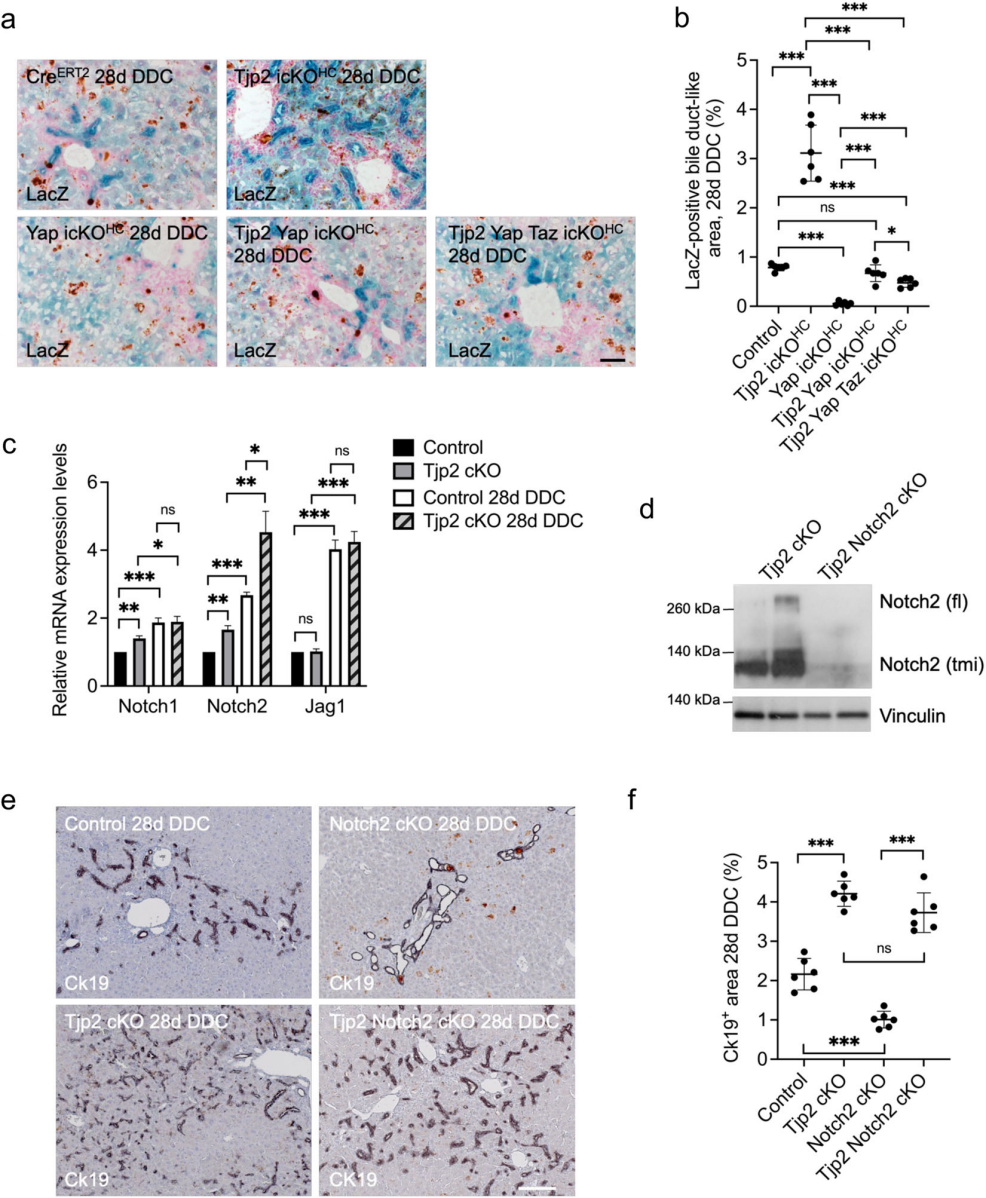
Inactivation of *Yap*/*Taz* but not Notch2 prevents the DDC-diet-induced hepatocyte to cholangiocyte transdifferentiation in *Tjp2* icKO^HC^ or *Tjp2* cKO mice. **a**, **b** LacZ staining of liver sections from the indicated mouse strains and quantification of LacZ-positive bile ducts. Scale bar: 100 μm. **c** Liver mRNA expression levels of Notch1, Notch2, and Jag1 of *Tjp2* cKO mice fed a normal or DDC diet and normalized to that of controls**. d** Western blot analysis of Notch2 to confirm deletion in the *Tjp2* Notch2 cKO liver. (tmi: transmembrane and intracellular domain, flL full-length). **e**, **f** Immunohistochemistry staining for Ck19 in liver sections from the indicated mouse lines and quantification. Scale bar: 100 μm. Data in (**b**, **c**, **f**) are shown as mean ± SD, unpaired Student’s *t* test. *p < 0.05; **p < 0.005, ***p < 0.0005, ns—not significant (*p* > 0.05), with *p* < 0.05 considered a significant difference. (**b**, **f**): *n* = 6, (**c**): *n* = 3 mice per cohort. icKO^HH^, tamoxifen-induced hepatocyte deletion.

Since DDC diet promotes the transdifferentiation of *Tjp2* deficient hepatocytes into Ck19-positive, *Tjp2*-negative cholangiocytes (see above), we next used lineage tracing to test if this transdifferentiation required Yap. LacZ-positive bile duct-like structures, indicative of transdifferentiation, were detected at a low frequency in livers from control DDC-diet fed Rosa26:LacZ mice but were only rarely observed in *Yap* cKO^HC^ Rosa26:LacZ livers (Fig. 7a, b). A large number of LacZ-positive biliary structures in *Tjp2* icKO^HC^ Rosa26:LacZ livers was strongly reduced after *Yap* inactivation, and concomitant inactivation of *Yap* and *Taz* resulted in a further reduction (Fig. 7b). Similar results were obtained for the different mouse strains by monitoring transdifferentiated bile ducts based on their Ck19-positive and Tjp2-negative phenotype (Fig. 4b, d).

Not only overexpression of Yap^2^, but also that of Notch2^1^ can induce transdifferentiation of hepatocytes into cholangiocytes. DDC-diet feeding increased Notch1, Notch2, and Jag1 mRNA expression levels in both control and *Tjp2* cKO livers (Fig. 7c). Interestingly, expression of Notch2 was higher in the liver of *Tjp2* cKO mice compared to controls, independent of whether they were fed standard chow or DDC-diet, suggesting an effect of Tjp2 on Notch2 expression. To test the possibility that the observed transdifferentiation in *Tjp2* cKO livers was mediated by the observed increase in Notch2 expression, we crossed *Tjp2* cKO and Notch2^F/F^ mice to generate *Tjp2* Notch2 cKO animals. As shown by Western blot and immunohistochemistry, expression of Notch2 was efficiently inactivated in the liver of these mice (Fig. 7d). While Notch2 cKO mice developed less bile ducts after a 4-week DDC diet compared to controls, *Tjp2* Notch2 cKO animals presented with a similar ductular reaction as observed in *Tjp2* cKO mice (Fig. 7e, f), indicating that the DDC-diet-induced ductular reaction in *Tjp2* cKO animals was not mediated by Notch2, and that the loss of Notch2 does not prevent the *Tjp2* deficiency related ductular reaction. Therefore, both Yap and Taz, but not Notch2, mediate the enhanced DDC-induced cholangiocyte transdifferentiation of hepatocytes lacking *Tjp2*.

## Discussion

TJP2-inactivating mutations in humans are associated with progressive familial cholestasis^9–11^. In the mouse, constitutive or inducible deletion of *Tjp2* in the liver only leads to mild progressive cholestasis, but these animals are more susceptible to dietary CA. While controls tolerate a diet supplemented with 0.5% cholic acid, *Tjp2* cKO mice develop severe cholestasis and liver injury^14^. Interestingly, we show here that mice lacking *Tjp2* in hepatocytes develop tolerance to a diet supplemented with DDC via an enhanced, DDC-induced, and Yap/Taz-mediated, transdifferentiation of hepatocytes into cholangiocytes.

*Tjp2* cKO mice fed a standard chow showed significantly higher plasma BA, AP, ALT, AST, and bilirubin levels, in agreement with previous data^14^ and consistent with mild cholestasis. When fed DDC diet for one week, these parameters were elevated as compared to control chow-fed cohorts, but, except for AP and bilirubin, comparable for DDC-diet-fed *Tjp2* cKO and control mice. After 4 weeks on the DDC diet, however, the *Tjp2* cKO mice presented with significantly less severe cholestasis and liver injury and better liver function as compared to corresponding controls, suggesting the development of tolerance to the detrimental effects of DDC. This adaptation was reflected in a robust expansion of Ck19, Sox9, and A6-positive, cholangiocyte-like cells. A ductular reaction in response to liver injury is a well-known phenomenon^24^, whereby liver progenitor cells and cholangiocytes in the periportal area expand. As expected, a ductular reaction was also observed in controls fed the DDC diet. Intriguingly, however, the DDC-diet-induced ductular reaction was not only more pronounced in the *Tjp2* deficient liver, but the duct-like structures were not restricted to the periportal area as in controls but were found further in the liver parenchyma. Additional bile ducts generated by this ductular reaction likely contribute to the tolerance of *Tjp2* cKO mice to prolonged DDC diet by facilitating clearance of toxic bile acids from the liver and relieving cholestasis. Consistent with the stronger ductular reaction in the Tjp2 cKO liver, cell proliferation of cholangiocytes, but not hepatocytes, was enhanced as compared to controls. However, using tamoxifen-inducible Albumin-Cre^ERT2^ and Sox9-Cre^ERT2^ mouse lines to inactivate *Tjp2* in hepatocytes or cholangiocytes, respectively^14^, revealed that the deletion of *Tjp2* in hepatocytes (e.g. *Tjp2* icKO^HC^ mice) and not in cholangiocytes (e.g. *Tjp2* icKO^CC^ mice) is responsible for the enhanced ductular reaction and the development of tolerance to the DDC-diet. A deletion of *Tjp2* in periportal “hybrid” hepatocytes, which express Sox9^25^ and can activate Sox9-Cre^26^, is thus unlikely to exclusively account for the observed ductular reaction.

The DDC-diet liver injury model has been widely used to study the transdifferentiation of hepatocytes into cholangiocytes^18,19^. Lineage tracing using a LacZ reporter confirmed abundant Ck19-positive ductular structures in the *Tjp2* icKO^HC^ liver that originate from LacZ-positive hepatocytes. In addition, since *Tjp2* is selectively ablated in hepatocytes (and not in cholangiocytes) in the *Tjp2* icKO^HC^ mouse, the fact that the DDC-diet-induced LacZ and Ck19-positive ductular cells were also devoid of Tjp2 further corroborates their origin from hepatocytes. In contrast, the more modest ductular reaction that was observed in DDC-diet fed control or *Tjp2* icKO^CC^ mice was predominantly derived from existing cholangiocytes.

Hepatocyte transdifferentiation is regulated by Hippo and Notch signaling^1,2^. Although the expression of several effectors of Notch signaling was upregulated in *Tjp2* cKO mice, deletion of Notch2 did not abrogate the DDC-diet-induced ductular reaction, suggesting that in this model, this process is independent of Notch2 signaling. Hippo signaling regulates the identity of cholangiocytes and overexpression of the Hippo effector Yap induces the transdifferentiation of hepatocytes into cholangiocytes^2^. Since pS127 Yap is targeted for degradation, the reduced pS127 Yap levels are consistent with the higher total Yap (and Taz) levels in Tjp2 cKO mice. Total Yap and active Yap (e.g. not phosphorylated at S127) levels were also increased in DDC-diet-fed *Tjp2* cKO and *Tjp2* icKO^HC^ mice compared to controls. While predominantly present at the hepatocyte cell border in DDC-diet-fed control mice, active Yap accumulated in the hepatocyte nucleus in the corresponding *Tjp2* icKO^HC^ animals.

Concomitant inactivation of *Tjp2* and *Yap*, *Taz*, or both, confirmed that the DDC-diet-induced ductular reaction in the absence of *Tjp2* was dependent on the Hippo effectors, with the deletion of *Yap* showing a more pronounced suppression of the ductular reaction. Although the increase in plasma BA and AP was less pronounced, blood and liver biochemistry parameters in *Tjp2 Yap* icKO^HC^ mice deteriorated to levels comparable to those in control or *Yap* icKO^HC^ animals. This observation indicates that the DDC-diet-induced ductular reaction in the absence of *Tjp2* may enhance the clearance of bile, thereby improving liver function and relieving cholestasis. Porphyrin plugs were still present in *Tjp2 Yap* icKO^HC^ livers, suggesting that the altered porphyrin clearance in the absence of *Tjp2* is *Yap* independent.

Although the inactivation of *Tjp2* in hepatocytes leads to stabilization and an increase of Yap and Taz protein levels, concomitant DDC exposure is required to induce the massive ductular reaction. In control mice, the DDC diet also induces a ductular reaction which, however, is limited and restricted to the periportal area. The excessive transdifferentiation of hepatocytes in *Tjp2* icKO^HC^ mice is suppressed by Yap deletion to levels comparable to control animals, with the residual ductular reaction now restricted to the periportal area. The limited periportal ductular reaction in control animals was also reduced after the deletion of *Yap* and, in both controls and *Tjp2* icKO^HC^ mice, was further suppressed after the additional inactivation of *Taz*. These observations indicate that both Yap and Taz contribute to hepatocyte transdifferentiation, that this is a physiological response to the DDC diet, and that Tjp2 negatively regulates this process. Yap and Taz directly interact via their C-terminal PDZ binding motifs with the 1^st^ PDZ domain of Tjp2 and mostly using MDCK cells, Tjp2 has been shown to repress Yap/Taz activity by preventing their nuclear translocation^4–8^. In vivo, such a regulatory role is less well established, but has been inferred from a correlation between reduced Tjp2/ZO-2 expression and increased Yap nuclear localization in a rat model of experimental compensatory renal hypertrophy and in liver steatosis of obese Zucker rats^7,8^. The present work thus establishes that Tjp2 negatively regulates Yap/Taz in vivo by suppressing DDC-induced hepatocyte transdifferentiation in the mouse. We postulate that the relative molar ratios of Yap and Tjp2 regulate Yap activity. Indeed, also overexpression of Yap in the liver can induce hepatocyte transdifferentiation^2^, possibly because the available Tjp2 can no longer retain the excessive Yap in the cytosol and/or present it to Lats1 for phosphorylation.

It is currently unclear if during transdifferentiation to cholangiocytes, hepatocytes transiently acquire an oval cell (e.g. A6-positive) identity. Yap has been implicated in the activation of oval cell proliferation and DDC-diet-fed mice showed an expansion of A6-positive cells^27^. While restricted to the periportal area in control mice, A6-positive cells spread over the entire liver in *Tjp2* cKO mice fed a DDC diet. Interestingly, despite higher Yap/Taz levels in the liver of *Tjp2* cKO mice, standard chow or CA^14^ supplemented diet did not result in the extra-periportal ductular reaction and hepatocyte transdifferentiation^14^ that was observed in *Tjp2* cKO mice fed a DDC-diet. These suggest that Yap/Taz activity or output are regulated or require additional factors, which themselves are modulated, directly or indirectly, by DDC or its metabolites. DDC activates the constitutive androstane receptor (CAR)^28^. Indeed, the CAR agonist TCPOBOP leads to nuclear accumulation of Yap in mouse hepatocytes^29^. CAR-mediated hepatocyte proliferation, but not the induction of drug-metabolizing enzymes, requires Yap^30^. DDC-diet-induced hepatic injury also activates a neonatal splicing program by suppressing the translation of Esrp2 mRNA, which rewires the Hippo pathway^31^. Although Tjp2 deletion and DDC diet feeding independently increase Yap protein levels, the contribution of both may be required to induce hepatocyte transdifferentiation. It will thus be interesting to further elucidate the crosstalk between Tjp2 and DDC in modulating Yap/Taz activity in the injured liver.

In summary, Tjp2 suppresses Yap-mediated transdifferentiation of hepatocytes into cholangiocytes in response to DDC-diet-induced liver injury. Mice with an ablation of *Tjp2* in hepatocytes develop tolerance to DDC-diet-induced liver injury through an enhanced, Yap dependent, transdifferentiation of hepatocytes to cholangiocytes. The resulting duct-like structures could contribute to the development of tolerance of these mice to the DDC diet, presumably by facilitating the clearance of bile from the liver.

## Methods

### Mouse strains, genotyping

Animal experimentation was approved by the relevant IACUC (A*STAR IACUC, Protocol #201558) and carried out under specific pathogen-free conditions. *Tjp2* was deleted in liver (Tjp2 Alb-Cre, Tjp2 cKO, liver-specific conditional knock-out), hepatocytes (Tjp2 Alb-Cre^ERT2^, after tamoxifen-induced deletion Tjp2 icKO^HC^), cholangiocytes (Tjp2 Sox9-Cre^ERT2^, after tamoxifen-induced deletion Tjp2 icKO^CC^)^14^. Mouse lines with tamoxifen-induced conditional deletion of the specified genes in hepatocytes (Alb-Cre^ERT2^) or cholangiocytes (Sox9-CreERT2) are referred to as “icKO^HH^” and “icKO^CC^”, respectively. Sox9-Cer^ERT2^ (C57BL/6-Sox9em1(cre/ERT2)Tchn/J) mice and the Rosa26:LacZ (Rosa26:Lox-STOP-Lox-LacZ) reporter line (B6;129S4-Gt(ROSA)26Sortm1Sor/J)^32^, used for lineage tracing, were obtained from Jackson laboratory. To delete *Yap* or WWtr1/*Taz* in hepatocytes, Tjp2^F/F^ and Alb-Cre^ERT2^ mice were crossed with Yap^F/F^ mice^23^ (generously obtained from Duojia Pan, Department of Molecular Biology and Genetics, Howard Hughes Medical Institute, Johns Hopkins University School of Medicine, Baltimore, USA, via Stefano Piccolo, University of Padova, Italy) and/or *Taz*^F/F^ mice^22^ (generously provided by Stefano Piccolo, University of Padova, Italy), or Notch2^F/F^ (Jackson Laboratory) mice to generate Yap^F/F^ Alb-Cre^ERT2^, *Taz*^F/F^ Alb-Cre^ERT2^, Tjp2^F/F^ Yap^F/F^ Alb-Cre^ERT2^, Tjp2^F/F^ *Taz*^F/F^ Alb-Cre^ERT2^, and Tjp2^F/F^ Yap^F/F^ *Taz*^F/F^ Alb-Cre^ERT2^ mice. Tamoxifen was used to delete the floxed *Tjp2*, *Yap*, *Taz*, or *Notch2* gene to obtain *Tjp2 Yap* icKO^HC^, *Tjp2 Taz* icKO^HC^ or *Tjp2 Yap Taz* icKO^HC^ mice, respectively. To delete *Notch2* in mouse liver, *Tjp2*^F^/^F^, Albumin-Cre mice, and *Notch2*^F/F^ (Jackson Laboratory) mice were crossed to generate *Notch2*^F/F^Alb-Cre and *Tjp2*^F/F^*Notch2*^F/F^Alb-Cre mice (*Notch2* cKO and *Tjp2 Notch2* cKO after gene deletions). To induce the deletion of the floxed alleles, 8-week-old mice were injected intraperitoneally (100 mg/kg) with tamoxifen (20 mg/ml in sunflower oil, Sigma-Aldrich) for 5 consecutive days and only used for further experimentation 7 days or later after the last tamoxifen injection. Littermate mice lacking Cre were used as controls and a previous characterization of other possible control mouse lines showed no significant differences among these in key parameters related to liver function and injury^14^. Male animals were used, but female mice showed comparable results. For genotyping, genomic DNA isolated from tail clippings was amplified using the following primer pairs to detect the wild-type or floxed alleles: *Yap*: P1: 5’-CCA TTT GTC CTC ATC TCT TAC TAA C-3’ & P2: 5’-GAT TGG GCA CTG TCA ATT AAT GGG CTT-3’ (wild-type: 498 bps, floxed: 597 bps); *Taz*: P1: 5’-TCT TCC AAG GTG CTT CAG AGA-3’ & P2: 5’-ATT TAG GCC AAA GTC GCT CA-3’ (wild-type: 238 bps, floxed: 312 bps); *Notch2*: P1: 5’-CAA CCC CAG ATA GGA AGC AG-3’ & P2: 5’-GAG CCT TTT CCC CAT ATT CC-3’ (wild-type: 202 bps, floxed: 240 bps). Genotyping for the *Tjp2* gene was done using primer-1 (5’-GTT CCT ATC CTG TTA GTT GGT AGT CC-3’) and primer-2 (5’-AAA GGG TCT CAT GTA GGT CAA GC-3’), yielding a 265 bp (wild-type allele) or a 422 bp (conditional mutant allele).

### Messenger RNA extraction and quantitative real-time polymerase chain reaction (qRT-PCR)

Total messenger RNA (mRNA) was extracted from whole liver and processed for quantitative reverse-transcription polymerase chain reaction using a QuantStudioTM 3 Real-Time PCR System (Applied Biosystems) and specific primers (Supplementary Table).

### Hepatocyte isolation and culture

Mice were anaesthetized using xylazine and ketamine as approved in the IACUC protocol. The abdomen was cut open and the inferior vena cava (IVC) was exposed. Perfusion with warm Kreb Ringer solution containing 50 mM EGTA was carried out using a catheter inserted into the mouse IVC. Once the blood was removed from the portal vein (PV) and the liver turned pale, the perfusion buffer was exchanged with digestion buffer containing Kreb Ringer solution with calcium chloride and Liberase^TM^ enzyme (Sigma). The digestion was carried out until the liver became soft and friable, indicating that digestion was completed. With the gall bladder removed, the liver was excised and transferred to a petri dish with the remaining digestion buffer to release the individual hepatocytes from the liver lobes. Liberase activity was inhibited by transferring the hepatocytes from the dish to a 50 ml tube containing culture media (RPMI containing 10% FCS and antibiotics) and the hepatocytes were gently washed twice with culture media. The number and viability of the hepatocytes were checked and cells plated for experiments.

### DDC diet

Mice were fed with chow diet supplemented with 0.1% 3,5-Diethoxycarbonyl-1,4-Dihydrocollidine (DDC: Cat #D80002, Sigma) for 28 days. When treated with tamoxifen, mice were provided with the DDC diet one week after the last tamoxifen dose.

### Serum and tissue biochemical analyses

Kits were used to determine bilirubin, serum alanine aminotransferase (ALT), alkaline phosphatase (AP) and aspartate aminotransferase (AST) (Teco Diagnostics), or plasma total BA (Diazyme

Laboratories) levels. For liver BA levels, 100 mg liver tissue was ground in liquid nitrogen, suspended in 1 mL water, sonicated, centrifuged, and BA levels were determined in the supernatant. Hydroxyproline was measured with the Hydroxyproline Assay Kit (Sigma, Catalog: MAK008). Briefly,10 mg liver tissue was homogenized and mixed with 100 µl water. Samples were hydrolyzed by the addition of 100 µl of concentrated (12 M) HCL for 3 h at 120 °C. After mixing and centrifugation at Mix and 10,000 x g for 3 minutes, the absorbance of the supernatant at a wavelength of 560 nm was measured. The numbers of mice per cohort analyzed are given in the respective figure legends, and samples were analyzed in 2 or 3 different and independent experimental runs.

### Immunofluorescence staining

Liver samples were embedded in OCT and sectioned at a thickness of 5 µm. Sections were stained with primary antibodies against ZO-2 (Tjp2) (rabbit; Cat #71–1400, Invitrogen, 1:100 dilution), Ck19 (rat; Troma III, Developmental Studies Hybridoma Bank DSHB, 1:20 dilution), A6 (rat; A6 BCM, DSHB, 1:20 dilution), Yap (rabbit; ab205270, Abcam, 1:50 dilution), HNF4α (mouse; MA1-199, Invitrogen, 1:100 dilution) and compatible fluorescently labeled secondary antibodies (Invitrogen, 1:200 dilution). Images were taken from at least 3 independent mice using a Zeiss LSM800 confocal microscope and Zen v3.4 (blue edition) software. Representative images are shown.

### Immunohistochemistry

Paraffin blocks were sectioned at a thickness of 5 µm. For immunohistochemistry, antigens were retrieved by steaming the slides for 20 min in a 2100 Retriever (Pick Cell Laboratories). The slides were then stained with primary antibodies against Ki67 (rabbit; Cat #9129 Cell Signaling, 1:100 dilution), Ck19 (rat; Troma III, DSHB, 1:20 dilution), A6 (rat; A6-BCM, DSHB, 1:20 dilution), Sox9 (rabbit; Cat #ab185230 Abcam, 1:50 dilution), Yap (mouse; Cat #12395 Cell Signaling, 1:50 dilution) and compatible fluorescently labeled secondary antibodies (Invitrogen, 1:200 dilution).

### Histology

Freshly dissected livers were fixed in 4% paraformaldehyde overnight, processed, and embedded in paraffin. 5 μm sections were stained with H&E or Sirius red and imaged with a Zeisscam

camera on a Zeiss Axio microscope. Five mice per cohort and images from at least 2 slides for each mouse were analyzed and used for quantification. Representative images are shown.

### LacZ staining

Livers were dissected seven days after the last tamoxifen injection, frozen in optimal cutting temperature compound, and 10 μm-thick sections cut and mounted on slides. After fixation in formalin for 10 minutes, LacZ staining was carried out (NovaUltra kit) per the manufacturer’s protocol.

Slides were counterstained with Nuclear Faster Red for 3–5 min. Three mice per cohort and at least 5 sections for each mouse were analyzed. Representative images are shown.

### Edu labeling

Edu, dissolved in DMSO (1 mg/10 µl) and further diluted in PBS (1 mg/100 µl), was injected intraperitoneally (1 mg/g body weight) 1 h before sacrificing the mice. Images were taken from at least 3 independent mice using a Zeiss LSM800 confocal microscope and Zen v3.4 (blue edition) software. Representative images are shown.

### Western blotting

Fresh liver samples were frozen in liquid nitrogen, crushed into powder and lysed for 15 min on ice in lysis buffer (50-mM Tris-HCl, pH7.5, 100 mM NaCl, 1 mM MgCl_2_, and 0.5% Triton X-100, supplemented with protease inhibitor cocktail and one PhosSTOP tablet per 10 ml [Cat.# 04 906 837 01, Roche]). Lysates were sonicated and centrifuged (13,000 × g for 15 min) at 4 °C. Supernatants were collected and equal amounts of protein were fractionated by SDS-polyacrylamide gel electrophoresis and subjected to Western blotting using antibodies against Alas-1 (rabbit; ab154860, Abcam), Alad (rabbit; ab151754, Abcam), Ck19 (rat; Troma III, DSHB), Collagen I (rabbit; Cat #NB600-408, NOVUS Biologicals), Hbms1 (rabbit; ab129092, Abcam), FC (mouse; sc377377, Santa Cruz), Laminin1-2 (rabbit; ab11575, Abcam), αSMA (rabbit; ab5694, Abcam), Yap/Taz (rabbit; Cat #8418, Cell Signaling), pSer127 Yap (rabbit; Cat #13008, Cell Signaling), Notch2 (rabbit; Cat #5732, Cell Signaling), Vinculin (mouse; Cat #V9131, Sigma). Primary and secondary antibodies were diluted 1:1000 and 1:3000, respectively. Samples from at least 3 independent mice were analyzed using ImageJ v15.3 and images of representative blots are shown. Gels and blots in a specific panel derive from the same experiment and were processed in parallel. Unprocessed and uncropped scans of blots shown in Figs. 1h and 5a are shown in Supplementary Fig. 3.

### Statistical analysis

Experimental design, the number of mice per cohort and replicates are described in detail under the respective methods and in the figure legends. The data, expressed as the mean ± SD, displayed normal distribution and variance between groups. Prism software (v. 9.3.1, GraphPad) was used for statistical analysis by unpaired Student’s *t* test. *p < 0.05; **p < 0.005, ***p < 0.0005, ns—not significant (*p* > 0.05), with *p* < 0.05 considered a significant difference.

### Reporting summary

Further information on research design is available in the Nature Research Reporting Summary linked to this article.

## Supplementary information

Supplemental Material

REPORTING SUMMARY

## Data Availability

The datasets generated during and/or analyzed during the current study are available from the corresponding author on reasonable request.
