# Supplementary material for: ZO-2/Tjp2 suppresses Yap and Wwtr1/Taz-mediated hepatocyte to cholangiocyte transdifferentiation in the mouse liver
Source: NPJ Regen Med. 2022 Sep 23;7:55. doi: 10.1038/s41536-022-00251-6 (PMC9508083; doi:10.1038/s41536-022-00251-6)
Supplement: Supplementary file 1 — Supplemental Material [file 41536_2022_251_MOESM1_ESM.pdf]

SUPPLEMENTARY FIGURES

Supplementary Figure 1

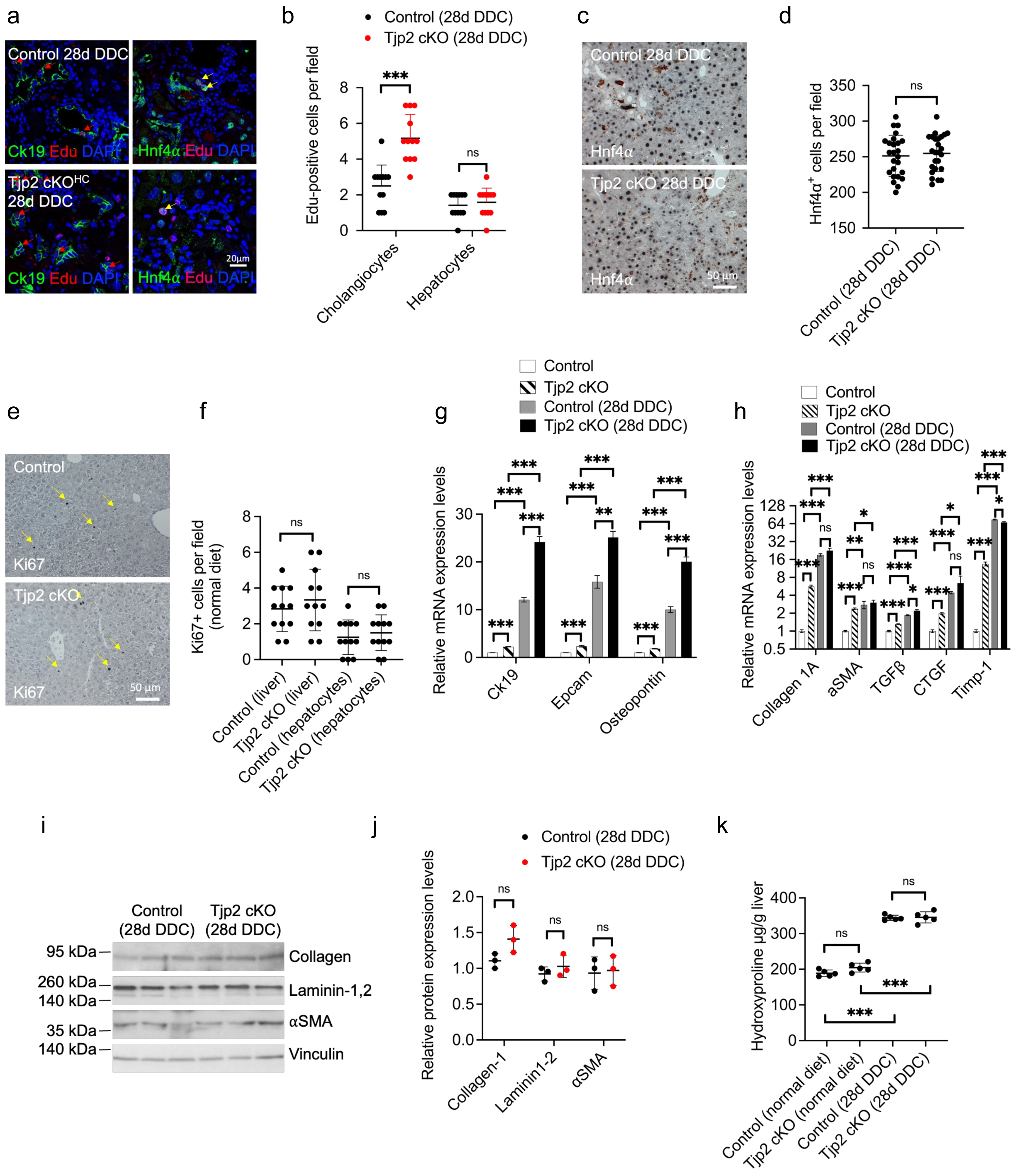

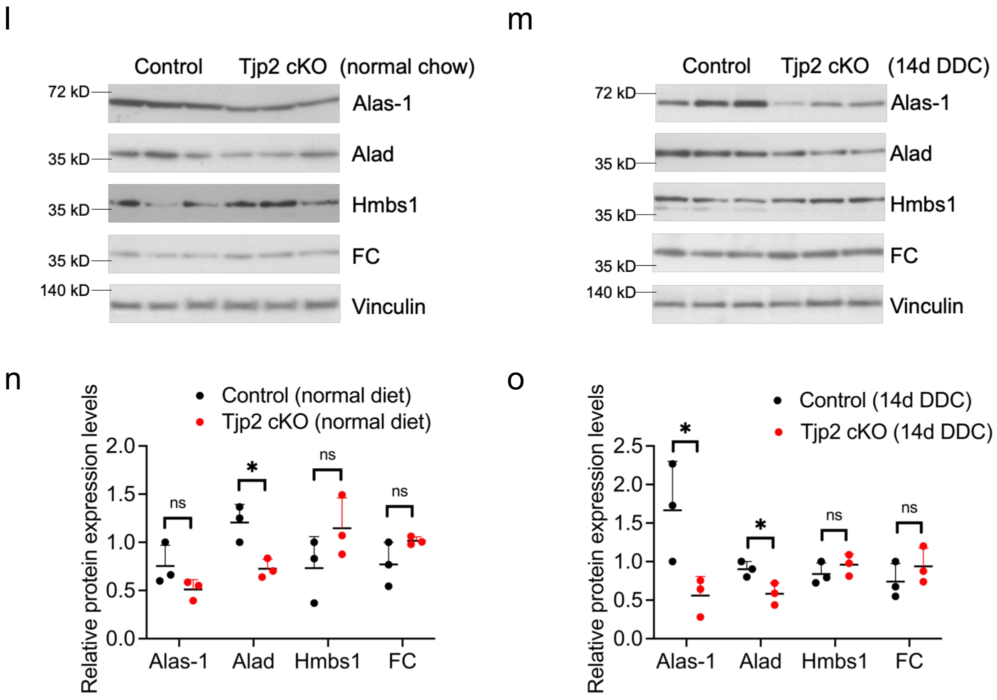

Supplementary Figure 1. Proliferation of hepatocytes and mRNA and protein expression of cholangiocyte and fibrosis markers in control and Tjp2 deficient mouse liver. (**a, b**) Immunofluorescence microscopy and quantification of Edu- and Ck19-positive cholangiocytes and Edu- and Hnf4α-positive hepatocytes in the liver of mice fed a DDC-diet for 28 days. Edu was injected 1 hour before sacrificing the mice. (**c, d**) Quantification of hepatocytes. Immunohistochemistry images (e) of liver sections of DDC-diet fed control or Tjp2 cKO mice were acquired and Hnf4α-positive hepatocytes in random fields counted and plotted (d). (**e, f**). Immunohistochemistry and quantification of Ki67-positive hepatocytes in the liver of mice fed a normal chow. (**g**). Relative mRNA expression levels of Ck19 and two of cholangiocytes growth markers, Epcam and Osteopontin analyzed by qRT-PCR. (**h**). qRT-PCR analysis of mRNA expression levels of fibrosis markers in livers of control and Tjp2 cKO mice fed a normal chow or a DDC-diet. (**i, j**). Protein expression detected by Western blot and quantification of fibrosis markers in livers of control and Tjp2 cKO mice fed a normal or a DDC-supplemented diet. (**k**) Quantitation of the fibrosis maker hydroxyproline in livers of control or Tjp2 cKO mice fed a normal chow or DDC-diet. (**l, m**) Western blot analysis of enzymes involved in hepatic porphyrin synthesis. Liver lysates from control and Tjp2 cKO mice, fed a normal or DDC-supplemented chow were blotted using antibodies to the indicated enzymes. Vimentin served as a loading control. (**n, o**). Quantification of protein expression levels in (k) and (l) normalized to vimentin expression. Note that aminolevulinic acid synthase 1 (Alas-1), the rate-limiting enzyme in hepatic porphyrin synthesis, is strongly downregulated in DDC-diet fed mice lacking Tjp2. Data in (b, d, f, g, h, j, k, n and o) are shown as mean ± SD, unpaired Student's t-test. *=p<0.05; **=p<0.005, ***=p<0.0005, ns=not significant (p>0.05), with p<0.05 considered a significant difference. (b, f): n=12, (d): n=25, (g, h, j, n and o): n=3, and (k): n=5 mice per cohort.

Supplementary Figure 2

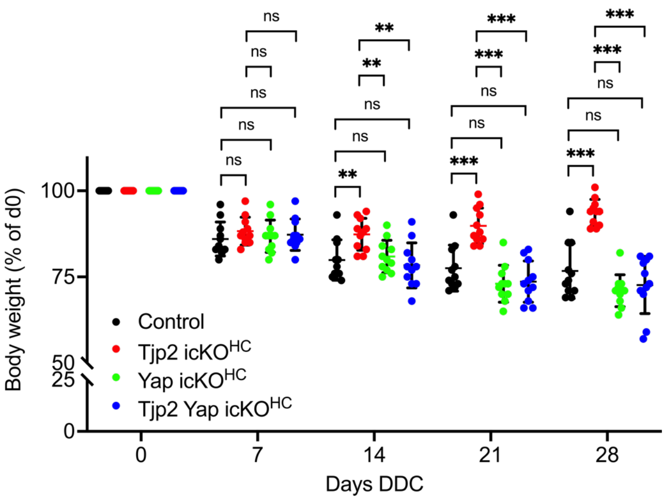

Supplementary Figure 2. Effect of Yap deletion changes in DDC-diet induced body weight changes of control and Tjp2 icKOHC mice. Changes in body weight relative of d0 are plotted for the indicated mouse lines after feeding a DDC-diet for 7, 14, 21 and 28 days. Data are shown as mean ± SD, unpaired Student's t-test. *=p<0.05; **=p<0.005, ***=p<0.0005, ns=not significant (p>0.05), with p<0.05 considered a significant difference. n=10 mice per cohort. icKOHH, tamoxifen-induced hepatocyte deletion.

Supplementary Figure 3

Fig. 1h

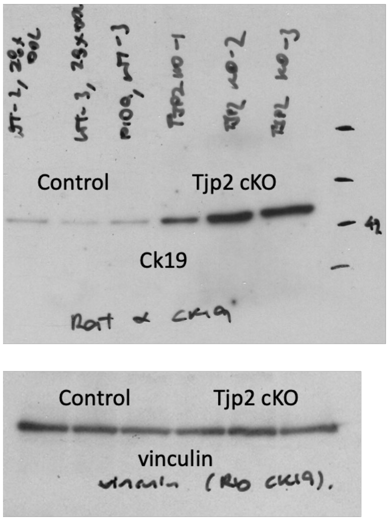

Fig. 5a

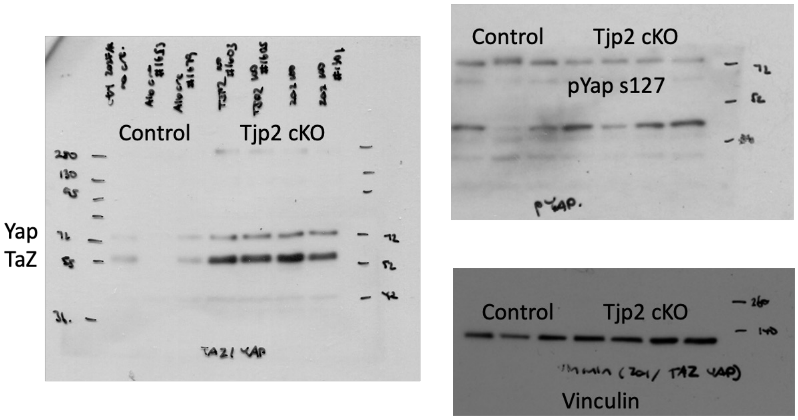

Supplementary Figure 3. Uncropped and unprocessed scans of blots shown in Fig. 1h and Fig. 5a.

SUPPLEMENTARY TABLE

Supplementary Table 1: Primer sequences used for qPCR analysis

Primers	Forward Primer Sequence (5' to 3')	Reverse Primer Sequence (5' to 3')
Yap	ATTTCGGCAGGCAATACGGA	TGCTCCAGTGTAGGCAACTG
Taz	AGGGATGGACTTCATTTTGGAGA	GGCTGTTAGGGAGGACATCATT
Sox9	GTGCAAGCTGGCAAAGTTGA	TGCTCAGTTCACCGATGTCC
Tead4	AACTCAAGTTTTGGCAAGGAGC	CAGGAGACTCAAAGCCTGGCA
Cyr61	TCCTGTCTTTGGCACCGAACCGCG	ACCAGGCGGCACTCTGGGTTGTCA
Notch1	TGTGGCTTCCTTCTACTGCG	CTTTGCCGTTGACAGGGTTG
Notch2	CAGCACTGTGACAGCCCTTA	TATTCCGCTCACAGGTGCTC
Jag1	AGGTACCTGCGTGGTCAATG	CACACAGGTCCCGCTATTGT
CK19	AGCGTGATCAGCGGTTTTG	CCTGGTTCTGGCGCTCTATG
EPCAM	GTCATTTGCTCCAAACTGGCG	AGCCCATCGTTGTTCTGGAT
Osteopontin	ACACTTTCACTCCAATCGTCC	TGCCCTTTCCGTTGTTGTCC
Collagen 1A	GACTGGAAGAGCGGAGAGTACTG	CAGGTCTGACCTGTCTCCATGTT
αSMA	CCTTCGTGACTACTGCCGAGC	TTCGTGGATGCCCGCTGACT
TGFβ	GCAGTGGCTGAACCAAGG	AGCAGTGAGCGCTGAATC
CTGF	ACATTAAGAAGGGCAAAAAGTGCA	AACTTAGCCCTGTATGTCTTCACA
Timp-1	GGCATCTGGCATCCTCTTGT	TAGCCCTTATGACCAGGTCCG
